# Bile Acids Quantification by Liquid Chromatography–Tandem Mass Spectrometry: Method Validation, Reference Range, and Interference Study

**DOI:** 10.3390/diagnostics10070462

**Published:** 2020-07-07

**Authors:** Elisa Danese, Davide Negrini, Mairi Pucci, Simone De Nitto, Davide Ambrogi, Simone Donzelli, Patricia M.-J. Lievens, Gian Luca Salvagno, Giuseppe Lippi

**Affiliations:** 1 Clinical Biochemistry Section, Department of Neurological, Biomedical and Movement Sciences, University of Verona, 37134 Verona, Italy; mairi.pucci@yahoo.it (M.P.); simone.denitto@studenti.univr.it (S.D.N.); gianluca.salvagno@univr.it (G.L.S.); giuseppe.lippi@univr.it (G.L.); 2 Department of Laboratory Medicine, University-Hospital of Padova, 35128 Padova, Italy; davide.negrini@studenti.unipd.it; 3 Waters S.p.a. Sesto San Giovanni, 20099 Milano, Italy; Davide_Ambrogi@waters.com (D.A.); Simone_Donzelli@waters.com (S.D.); 4 Experimental Biology Section, Department of Neurological, Biomedical and Movement Sciences, University of Verona, 37134 Verona, Italy; patricia.lievens@univr.it

**Keywords:** bile acids, LC–MS/MS, interference

## Abstract

Bile acids (BA) play a pivotal role in cholesterol metabolism. Their blood concentration has also been proposed as new prognostic and diagnostic indicator of hepatobiliary, intestinal, and cardiovascular disease. Liquid chromatography tandem mass spectrometry (LC–MS/MS) currently represents the gold standard for analysis of BA profile in biological samples. We report here development and validation of a LC–MS/MS technique for simultaneously quantifying 15 BA species in serum samples. We also established a reference range for adult healthy subjects (*n* = 130) and performed a preliminary evaluation of in vitro and in vivo interference. The method displayed good linearity, with high regression coefficients (>0.99) over a range of 5 ng/mL (lower limit of quantification, LLOQ) and 5000 ng/mL for all analytes tested. The accuracies were between 85–115%. Both intra- and inter-assay imprecision was <10%. The recoveries ranged between 92–110%. Each of the tested BA species (assessed on three concentrations) were stable for 15 days at room temperature, 4 °C, and −20 °C. The in vitro study did not reveal any interference from triglycerides, bilirubin, or cell-free hemoglobin. The in vivo interference study showed that pools obtained from hyper-cholesterolemic patients and hyper-bilirubinemic patients due to post-hepatic jaundice for benign cholestasis, cholangiocarcinoma and pancreatic head tumors had clearly distinct patterns of BA concentrations compared with a pool obtained from samples of healthy subjects. In conclusion, this study proposes a new suitable candidate method for identification and quantitation of BA in biological samples and provides new insight into a number of variables that should be taken into account when investigating pathophysiological changes of BA in human diseases.

## 1. Introduction

Bile acids (BA) are amphipathic end products of cholesterol metabolism. Primary BA (cholic acid CA, chenodeoxycholic acid CDCA) are synthesized in the liver through oxidation of the precursor molecule cholesterol. After conjugation with either taurine or glycine, conjugated BA are secreted into the gallbladder and subsequently pass into the small intestine via bile duct [1]. Along the terminal ileum, nearly 95% of BA are reabsorbed and transported back to the liver via the portal vein, where they are taken up by hepatic BA transporters, re-conjugated, and re-excreted into the bile through enterohepatic circulation [2]. However, a certain proportion of BA reaches the colon and undergoes extensive microbial transformations until conversion into the so-called “secondary BA” (e.g., deoxycholic acid (DCA), ursodeoxycholic acid (UDCA), and lithocholic acid (LCA)) [3].

Although BA have been traditionally considered digestive compounds, whose main function is helping in emulsion and absorption of dietary fats and liposoluble vitamins [4], recent findings suggested their participation in a broader array of physiologic processes, including energy expenditure, bile flow, intestinal motility, bacterial growth, and inflammation. More importantly, BA have garnered enhanced interest for their potential involvement in cancer development and progression [5,6]. In previous studies, physiological and pathophysiological changes in circulating BA profiles have been associated with development of degenerative liver and intestinal diseases, chronic inflammation, gut barrier dysfunctions, cholestasis, and colon cancer [7,8]. Accordingly, serum or plasma BA composition patterns have been proposed as new diagnostic or prognostic tool in various liver diseases [9,10,11,12]. 

Nonetheless, the measurement of BA in biological fluids is not an easy enterprise. Conventional methods consist of enzymatic techniques based on the use of dehydrogenases acting on hydroxyl groups in BA structures, and thus allowing quantification of total BA [13]. Their use is of limited value when comparisons are made between populations in which BA differ not necessarily in total concentration, but rather in relative percentages. The next generation methods for BA analysis are most likely based on mass spectrometry (MS). Considering the close structural similarity of isobaric BA isomers in biological samples, coupling to chromatographic separation is usually required. To date, liquid chromatography–tandem mass spectrometry (LC–MS/MS) techniques are considered the gold standard for analysis of BA profile in both bile and serum samples [14]. These methods provide exceptional resolution, sensitivity and specificity. However, simultaneous measurements of all BA into a single LC–MS/MS run is complex, and only few research groups worldwide have implemented this approach. Another aspect that has been scarcely appreciated so far is the close dependence of BA levels on those of cholesterol, their precursor molecule. It is indeed reasonable to suppose that hyper-cholesterolemic patients might show impaired BA metabolism compared to normo-cholesterolemic subjects. Moreover, obstructive cholestasis is known to result in accumulation of BA and bilirubin in the circulation. Since BA are implicated in the pathogenesis of cholestatic liver damage through mechanisms involving oxidative stress, it is likely to assume that non-jaundice patients might show a BA profile different from that of patients with jaundice due to benign biliary disease, and even more different from those with malignant biliary obstruction.

We herein report the development and validation of a LC–MS/MS method to simultaneously quantify 15 BA species in serum samples. As part of standard protocols used in our laboratory practice we also performed an experimental in vitro study aimed at exploring whether increasing values of triglycerides, bilirubin, or cell-free hemoglobin may have an impact on accuracy and precision of LC–MS/MS quantification due to matrix effect or other types of interference. We then explored, for the first time, the influence of hyperbilirubinemia and hypercholesterolemia in vivo. Finally, reference ranges for single BA in a healthy adult population were established.

## 2. Materials and Methods

### 2.1. Analytical Standards and Solvents

Tauroursodeoxycholic acid (TUDCA), taurocholic acid (TCA), glycoursodeoxycholic acid (GUDCA), glycocholic acid (GCA), taurochenodeoxycholic acid (TCDCA), taurodeoxycholic acid (TDCA), cholic acid (CA), ursodeoxycholic acid (UDCA), glycochenodeoxycholic acid (GCDCA), hyodeoxycholic acid (HDCA), glycodeoxycholic acid (GDCA), taurolithocholic acid (TLCA), chenodeoxycholic acid (CDCA), glycolithocholic acid (GLCA), deoxycholic acid (DCA), taurocholic acid-d4 (d4-TCA), glycocholic acid-d4 (d4-GCA), cholic acid-d4 (d4-CA), ursodeoxycholic acid-d4 (d4-UDCA), chenodeoxycholic acid-d4 (d4-CDCA), and deoxycholic acid-d4 (d4-DCA) were purchased from Sigma–Aldrich (St. Louis, MO, USA) or Cayman Chemical (Ann Arbor, MI, USA). LC/MS grade methanol, acetonitrile, isopropanol, ammonium formate, and formic acid were purchased from VWR International (Radnor, PA, USA)

### 2.2. Calibrators, Quality Control Samples, and Internal Standard Solution

Calibrators and quality control (QC) samples were prepared from a methanolic stock solution containing a final concentration of 5000 ng/mL of each standard. Calibrators were prepared by diluting the methanolic stock solution to eight levels (5 ng/mL, 12.5 ng/mL, 25 ng/mL, 50 ng/mL, 250 ng/mL, 1250 ng/mL, 2500 ng/mL, 5000 ng/mL) with both methanol or synthetic serum (Steroid Free Serum, ChromSystems, Gräfelfing, Germany). Quality controls were prepared by diluting the methanolic stock solution to three levels (18.75 ng/mL, 125 ng/mL, 1875 ng/mL) in methanol. The internal standards mix solution was prepared in methanol, with final concentration of 10 µg/mL for each of the five deuterated BA. Preparations of calibrators and quality controls followed the same preparation procedure of samples.

### 2.3. Sample Collection

Blood samples were collected into tubes with clot activator and gel separator (Vacutest, KIMA, Padova, Italy) and centrifuged within 30 min from collection. Serum samples were then separated, aliquoted, and kept frozen at −80 °C until analysis. All participants provided an informed consent for participation to this study, which was carried out in accordance with the Declaration of Helsinki and was approved by the local ethical committee (University Hospital of Verona; 971CESC, 25 July 2016 and 24113CESC, 16 May 2017).

### 2.4. Sample Preparation

An aliquot of 200 µL of serum sample was spiked with 780 μL methanol and 20 μL of the internal standard solution described above in plastic centrifuge tubes. Samples were vortexed for 20 s, centrifuged for 5 min at 18,000 rcf, and then 200 µL of the supernatant was transferred in a 96-wells plate for analysis along with 200 µL of water.

### 2.5. LC–MS/MS Settings

Chromatographic separation was performed using an Acquity UHPLC I-Class System FTN system. Samples in the same analytical session were kept in the autosampler at 10 °C until analysis. The injection volume was always 10 µL. The chromatographic column was a Cortecs T3 2.7um (2.1 × 30 mm), kept at 60 °C. The mobile phase A was composed by water, 0.1% of 200 mM ammonium formate, 0.01% of formic acid. The mobile phase B was composed by half acetonitrile and half isopropanol, 0.1% of 200 mM ammonium formate, 0.01% of formic acid. The chromatographic run for each sample was 7-min long, with a total of 1 mL/min flow during the gradient. Starting with 5% phase B at time zero, we set a linear increase until minute 5.5 with 50% phase B, then from 5.5 to 6.2 min set 98% phase B and then from 6.2 to 7.0 min the column reconditioning with 5% phase B. Quantification was performed using a Xevo TQ-S micro MS/MS detector with multiple reaction monitoring mode (MRM) and electrospray negative ionization mode (ESI). Source-specific and compound-specific parameters were optimized to obtain maximal signal response with direct infusion. Common settings for all compounds were the following: capillary voltage 2 kV, cone voltage 60 V, source temperature 150 °C, desolvation temperature: 600 °C, cone gas flow 50 L/h, desolvation gas flow 1000 L/h. Data were collected and analyzed using MassLynx V4.2 SCN977.

### 2.6. Validation Procedures

Linearity was evaluated using the calibration curve in methanol and in the steroid-free serum with linear regression and 1/x weighting. The lowest limit of quantitation (LLOQ) was calculated as the lowest level from the linearity study with a precision less than 20%. Accuracy and precision were tested with quality control (QC) samples prepared from the standard stock solution at three concentrations (see above). All QC samples were pre-treated and measured as standard curve samples, with six replicates for each of the three levels. Accuracy was estimated as average percentage difference between measured and expected concentration. Precision was calculated as coefficient of variation (CV%) of measured concentrations. Intra-assay imprecision was assayed with 15 replicates per level, whilst inter-assay imprecision was assayed with 10 replicates per level in 10 consecutive days. 

Apparent recovery was evaluated by calculating the ratio of the areas under the curve (AUCs) of each analyte in a sample spiked before and after the precipitation procedure.

Matrix effect was evaluated by comparing the AUCs of each calibration point and compound (divided by the AUC of their internal standard) of calibration curve and linearity study in methanol and in the steroid-free serum. Stability studies were conducted with quality control samples and two pools of serum samples, kept at room temperature (RT), 4 °C, and −20 °C for up to 15 days. Finally, carryover effect was evaluated by injecting methanol after the injection of the most concentrated calibrator.

All analytical procedures were carried out according to the CLSI C62-A guideline [15].

### 2.7. Reference Ranges

To establish definitive reference ranges in adults, samples were collected from 130 healthy blood donors and analyzed (66 male, 64 females; median age: 45 years old, range: 18–70 years).

### 2.8. Interference Study

Total and conjugated bilirubin concentrations were measured in each aliquot on a Roche Cobas 8000 (colorimetric diazo method; Roche Diagnostics, Risch-Rotkreuz, Switzerland). Triglyceride and total cholesterol concentrations were measured in each aliquot on a Roche Cobas 8000 (enzymatic colorimetric test; Roche Diagnostics, Risch-Rotkreuz, Switzerland). The hemolysis degree was assayed in all aliquots as hemolysis index (H-index) on a Roche Cobas 8000 (Roche Diagnostics, Risch-Rotkreuz, Switzerland), as described elsewhere [16].

#### 2.8.1. In Vitro Study

The first plasma pool displaying baseline triglyceride concentration of 1.67 mmol/L was spiked at fixed ratio with increasing concentrations of exogenous triglycerides (SMOFlipid 200 mg/mL; Fresenius Kabi, Verona, Italy) up to 11.35 mmol/L. The second pool displaying baseline total bilirubin concentration of 9.5 μmol/L was spiked at fixed ratio with increasing total bilirubin concentrations (Product Number B 4126; Sigma–Aldrich, Saint Louis, MO, USA) up to 658 μmol/L. The third hemolytic pool was generated by mechanical injury of red blood cells, by passing whole anticoagulated blood through a very fine needle, as described elsewhere [17]. Scalar amounts of this hemolyzed blood were then added to the non-hemolyzed pool to obtain gradually increasing values of cell-free hemoglobin in plasma, to yield a final concentration comprised between 0.03 g/L (i.e., non-hemolyzed blood) and 4.1 g/L. A detailed procedure for serum pool preparation has been previously described [18].

#### 2.8.2. In Vivo Study

A total of 20 patients with total cholesterol >8.00 µmol/L were collected, pooled together, and then diluted with a pool from patients with total cholesterol <2.5 µmol/L. Similarity, to evaluate in vivo bilirubin interference, three pools of plasma obtained from patients with post-hepatic jaundice were diluted with a pool from obtained from patients with serum conjugated bilirubin value <5 µmol/L. Three cholestatic pools were also obtained by mixing equal volumes of serum samples collected from (a) 20 patients with biliary stones, (b) 20 patients with cholangiocarcinoma, and (c) 20 patients with pancreatic head tumors. Levels of direct bilirubin in the three pools were 5.1 µmol/L, 139 µmol/L, and 164 µmol/L respectively.

## 3. Results

### 3.1. LC–MS/MS Optimization

**Figure 1:**
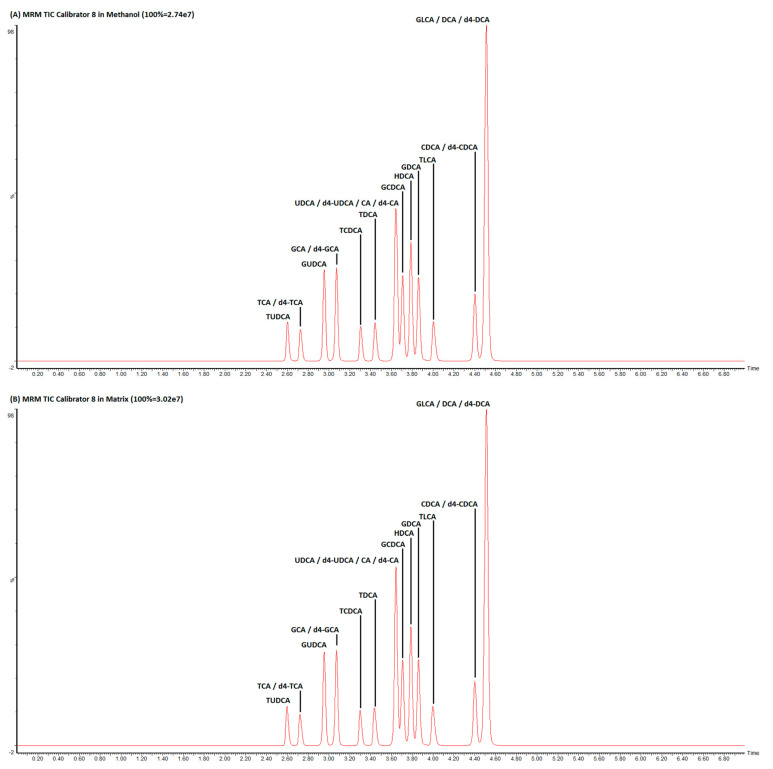
Representative chromatographic run of a calibrator in methanol (**A**) and synthetic serum (**B**).

**Figure 2:**
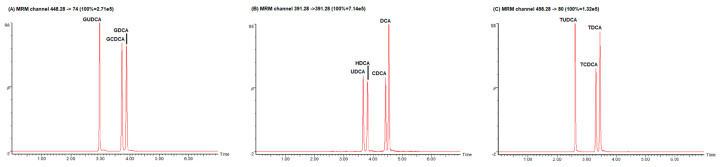
Chromatographic separation of three groups of isobar compounds. (**A**) Isobaric compound: GUDCA, GCDCA, GDCA; (**B**) Isobaric compounds: UDCA, HDCA, CDCA, DCA; (**C**) Isobaric compounds: TUDCA, TCDCA, TDCA. Isobaric compounds (i.e., compounds that share mass but are structurally unrelated) can contribute to errors even with the high specificity afforded by tandem mass spectrometry. Figure shows that the LC–MS/MS method set up in our laboratory is able to separate isobars with high peaks resolution.

Our proposed UHPLC–MS/MS (Ultra High Performance Liquid Chromatography–tandem mass spectrometry) technique would enable the quantification of 15 different bile acid species from 200 μL of serum samples, with an analytical run time of 7 min, and was set up after slightly modifications of an existing application note developed by Waters (Waters Application Note 720006261EN “MetaboQuan-R for Bile Acids in Human Serum: A Rapid, Targeted UHPLC–MS/MS Method for Metabolomics Research Studies” from Billy Joe Molloy Wilmslow UK). A representative chromatographic run showing BA separation is shown in Figure 1. Optimal source-specific parameters and compound-specific parameters (Table 1) were determined with direct infusion of standard compounds. The most abundant fragment ions were chosen for quantification purpose. Due to the lack of specific fragment ions, quantification of some unconjugated BA (UDCA, HDCA, CDCA, DCA) was accomplished by monitoring the same *m/z* values for both the precursor ion and the fragment ion, consistent with published literature [19]. LC conditions were optimized to achieve an optimal separation of three isobar groups of BA (Figure 2).

### 3.2. Method Validation

The assay was linear both in methanol and in matrix for all BA species tested over a dynamic concentration range between 5 ng/mL and 5000 ng/mL with a coefficient of determination (*r*^2^) summarized in Table 1. The lower limit of quantification (LLOQ) of each bile acid species were determined to be the lowest point in the linearity study (5 ng/mL). No lower concentrations were evaluated. The accuracies were between 85% and 115%. Both intra- and inter-assay CVs were <10% for all levels tested. The recoveries ranged between 92% and 110% (mean 95.5%). No carryover effect could be identified. No matrix effect could be detected; the difference between AUCs (divided by their internal standard AUC) of the calibration curves in methanol and in the steroid-free serum of all BA that we have investigated was always found to be <10%. Each of the tested BA species (three levels) were stable for 15 days at room temperature, 4 °C, and −20 °C (results were within 10% from the originally measured concentrations).

### 3.3. Reference Ranges

The mean of total BA in our adult healthy population was 795.4 ng/mL (95% CI: 171.9–1965.8 ng/mL). The reference intervals of unconjugated and glycine and taurine conjugates were as follows: 374.5 ng/mL (95% CI: 37.3–1128.4 ng/mL), 409.6 ng/mL (95% CI: 72.5–1217.8 ng/mL), and 74.98 ng/mL (95% CI: 22.5–195.9 ng/mL) respectively. Reference ranges of individual BA are shown in Table 2. For the calculations, values below the lower LOQ were imputed as LOQ/sqrt(2) [20].

### 3.4. Interference Study

#### 3.4.1. In Vitro Study

In vitro hyper-triglyceridemia, hyper-bilirubinemia, and hemolysis did not interfere with BA quantification. The CV% from scalar dilutions were all within the intra-assay imprecision of the method.

#### 3.4.2. In Vivo Study

**Figure 3:**
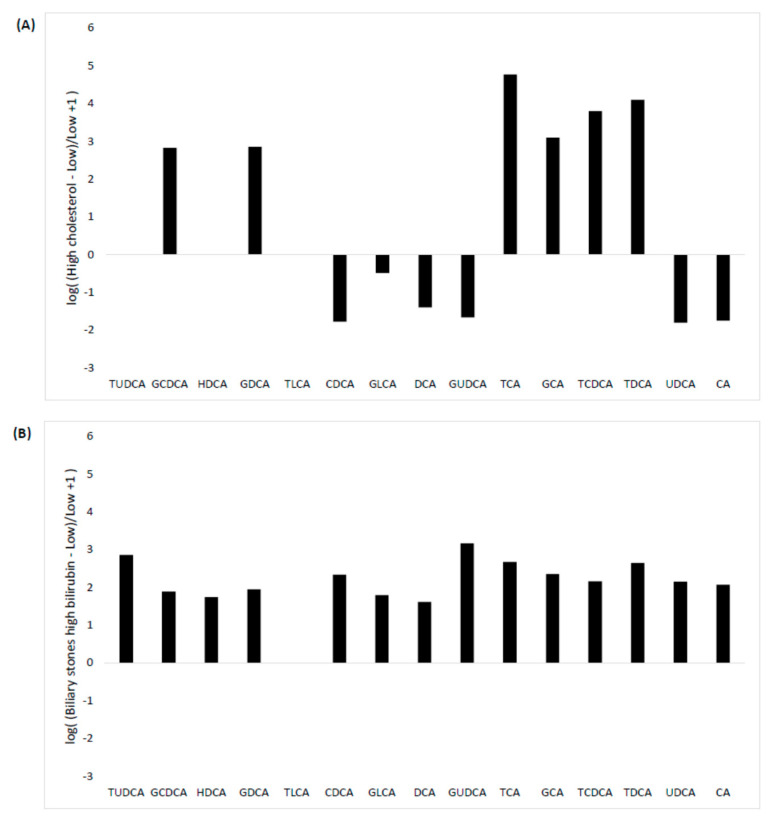

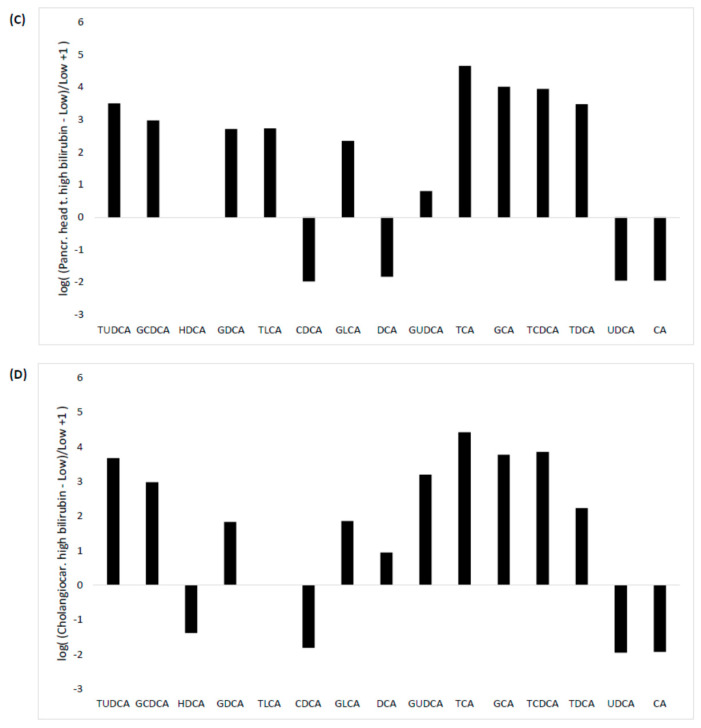
Influence of hyper-cholesterolemia and hyperbilubinemia on BA profile. Comparison between the hyper-cholesterolemic pool and the pool from normo-cholesterolemic subjects (**A**). Comparison between the normo bilirubiunemic pool and the hyperbilirubinemic pools from biliary stones (**B**), pancreatic head tumor, (**C**) and cholangiocarcinoma (**D**) patients.

The linearity of the assay was found to be well maintained for all the considered BA (*r*^2^ values between 0.972 and 0.999) in the hyper-cholesterolemic and the three hyper-bilirubinemic pools dilutions. The difference in BA concentration obtained by comparing serum pools of hyper-cholesterolemic patients and those with post-hepatic jaundice with pool obtained from healthy subject is shown in Figure 3. The comparison between the hyper-cholesterolemic pool and the pool from normo-cholesterolemic subjects showed a great increase for all the most represented tauro-conjugated BA (excluding TLCA which was always below the LLOQ), and a two-fold reduction in all un-conjugated BA (Figure 3A). In our experimental conditions the increase in tauro-conjugated BA was found to be nearly 65-fold for TCDCA, 128-fold for TDCA, and 588-fold for TCA, respectively. High levels of serum bilirubin levels demonstrated a different impact on BA concentrations depending on the underlying causes of jaundice. BA concentrations were slightly affected by increased bilirubin levels due to benign condition (i.e., biliary stones). From the comparison between post-hepatic jaundice from biliary stones and normo-bilirubinemic pool we found a relatively modest 2-fold increase in total BA concentration. TUDCA and GUDCA were the two BA showing the greatest increase in pool from biliary stone patients compared to normal pools (i.e., 12- and 16-fold increase, respectively) (Figure 3B).

On the other hand, post-hepatic jaundice due to malignant tumors had a larger impact on the BA profile. Except for DCA, which showed an inverse trend in cholangiocarcinoma and pancreatic head tumor (a slight 3-fold increase in pancreatic head tumor and a slight one-fold decrease in cholangiocarcinoma compared to normal pool), both tumors displaying an increase in all the tauro- and glyco-conjugated BA and decreased CA, CDCA, and UDCA (Figure 3C,D). TCA was found to be the most affected BA, showing a 457-fold increase in the pool of serum samples collected from pancreatic head tumor patients and a 267-fold increase in that obtained from cholangiocarcinoma patients compared with the pool of serum samples collected from healthy individuals. Irrespective of comparable confrontable conjugated bilirubin values measured in the serum pools from patients with cancer, total BA levels showed a greater increase in the pool of serum samples obtained from patients with pancreatic head tumor (22-fold increase compared to normal pool) than the total BA increase observed in cholangiocarcinoma patients (14-fold increase compared to normal pool).

## 4. Discussion

In recent years, LC–MS/MS has become the most suitable method for the direct and simultaneous quantitative profiling of free and conjugated BA in biological samples, thus including plasma, serum and bile. We thus report here the development and validation of a quantitative LC–MS/MS method for BA profiling which uses a very modest sample volume, requires easy sample preparation, and encompasses a short chromatographic run. It is characterized by a LOQ of 5 ng/mL and a good linearity between 5 and 5000 ng/mL for all compounds tested. The wide measurement range, the good linearity, the lack of matrix effect from known interference substances, good recovery, accuracy, and precision along with the lack of carry-over persuaded us to conclude that this method may be seen as a valuable tool for applications in ongoing clinical studies.

The analytical technique described herein represents a substantial development beyond the kaleidoscope of methods currently reported in the scientific literature. First, it is characterized by considerably short chromatographic run (i.e., only 7 min), i.e., much shorter compared to those published so far, which need between 15–45 min [21,22,23,24,25,26]. A comparable turnaround time has already been published, but only limited to the analysis of a small subset of BA [27].

Unlike other methods of preparation, in which proteins are separated from serum by extraction, precipitation or filtration, the “dilute-and-shoot” method developed herein dilutes and solubilizes the proteins throughout the chromatographic portion of the analysis. By featuring chemical protein precipitation, removal of proteins by centrifugation and direct analysis of supernatant, this approach eliminated the potential for BA loss due to pre-processing steps such as solid phase extraction and the commonly applied procedure of sample drying prior to reconstitution with water [27], methanol [8], or their combination [28]. In such way, selective enrichment or exclusion of BA species across a wide range of hydrophobicity has been minimized or entirely avoided. The dilute-and-shoot approach currently represents the fastest and simplest option for high throughput measurements, and it guarantees good accuracy, precision and linearity in a wide range of clinical applications [29], including BA quantification in plasma samples [26]. The extraction efficiency of our method has been confirmed by evidence of high recovery rates, between 115–133%. The relative low sensitivity is a possible limitation of this approach. Recently published methods providing concentration of samples by nitrogen-drying achieved detection limit below 1 ng/mL for most BA, which is >10-fold lower than the LOQ that can be achieved with our assay [30]. However, due to the purpose of obtaining a suitable method for future clinical studies, sensitivity has been outweighed by fast and high throughput analyses.

Finally, our study is the first to establish a BA definitive reference range in a Caucasian Italian adult population. Another study, which earlier investigated the reference ranges in different ethnic groups, has shown that the median values of adult Caucasians were almost overlapping with our data [31]. Conversely, a more recent investigation, including 20 healthy individuals [11], found that the median values of most BA were higher than those observed in our study. Nonetheless, this difference could be attributable to the different age of the population, which has not been clearly reported in the previous investigation of Salihović et al. [30]. Along with the validation procedure and establishment of reference ranges, we also performed interference studies aimed at assessing the matrix effect from in vitro hemolysis, lipemia, and hyper-bilirubinaemia. These three types of interference are known to impact the performance of optical methods due to visual color and turbidity changes, but can also impair precision and accuracy of LC–MS/MS methods. In particular it has been reported that human hemoglobin or serum protein released by the erythrocytes injuries may cause ion suppression when eluting at the retention time near to that of the components under evaluation [31]. In our in vitro study we failed to identify any difference in instrument response between test and control samples, whereby neither ion suppression/enhancement nor interference from any of the spiked substances could be recorded.

Even more interestingly, we performed an interference study for exploring the effect of in vivo hyper-bilirubinemia and hyper-cholesterolemia. BA are known to play a major role in cholesterol homeostasis. Hyper-cholesterolemia causes increased hepatic sterol 27-hydroxylase activity and alternative BA synthesis, thus determining a general expansion of BA pool [32]. Hence, increased total serum cholesterol is likely to affect not only total serum BA concentration but also individual BA metabolism. According with this hypothesis, by comparing a pool of serum samples collected from hyper-cholesterolemic patients with that obtained from normo-cholesterolemic subjects, we found a significant difference in most of the BA tested. Along with a clear increase in tauro-conjugated BA we also observed a slight decrease in non-conjugated BA such as CDCA, UDCA, CA, and UDCA in the pool of hyper-cholesterolemic serum samples. Interestingly, a recent study which assessed fecal BA and blood lipid profile of male volunteers with hyper-cholesterolemia and normo-cholesterolemia failed to find significant difference in BA concentrations between these two groups, though a trend toward increased DCA and CA was observed in the former cohort of patients [33]. Another retrospective follow-up study revealed that in total BA excretion, DCA and LCA were positively related to high density lipoprotein cholesterol level and were found to be an independent risk factor for ischemic stroke [34]. Our preliminary finding highlights the need to take into account not only differences in body mass index [35] and dietary changes [36], as already described, but also cholesterol levels when investigating pathophysiological changes of BA.

A number of studies have also evaluated the association between BA composition and/or concentration and post-hepatic jaundice by biliary obstruction, although the causal relationship is still unclear. What is certain, however, is that the biological basis of benign and malignant biliary stenosis differs significantly and that BA may somehow play a role. Accordingly, when using BA serum profile as potential biomarkers in human diseases it is important to take into account that, in patients with increased bilirubin levels, BA profile varies according to the specific causes of jaundice. By comparing the BA individual concentrations from serum pools from patients with different causes of post-hepatic jaundice with those from normo-bilirubinemic subjects, different patterns of BA could be clearly observed. More specifically, we found a general, modest increase of almost all BA in benign obstruction and an often-considerable variation of in conjugated vs. non-conjugated BA in samples collected from both cholangiocarcinoma and head-pancreas malignant tumours patients. Some trends found here are in keeping with data from case controls studies earlier published [11,14,37], though real comparisons cannot be made due to the different study design. Interestingly, although no data have been published so far on BA serum profile in patients with tumors of the pancreatic head, the unconjugated BA CA, CDCA, and UDCA that were found to be decreased in our serum pool were the same that were instead reported to be increased by Rees and co-authors in bile samples from the same type of cancer [38]. These preliminary finding pave the way for better characterization of BA serum and biliary profiles in patients with pancreatic cancer.

Since the first mass spectral analyses of BA, the state of the art has advanced steadily so that the current generation of laboratory instrumentation is fully capable of separating and identifying specific metabolites in the nanograms range [39]. Contextually, clinical studies demonstrating the role of BA as new biomarkers in different liver and non-liver diseases is growing fast.

This study attempts to propose an innovative candidate method for identifying and quantifying BA in clinical studies, providing also a comprehensive understanding of BA profiles in healthy subjects, and ultimately highlighting the need to consider the influence of cholesterol and bilirubin abnormalities when investigating serum BA profiles in patients with many different diseases.

## Figures and Tables

**Table 1:** Compound specific parameters and linearity results for each bile acid species.

Type	Bile Acid	Internal Standard	Precursor Ion *m/z*	Retention Time (min)	Product Ion *m/z*	Collision Voltage (V)	Calibration Linearity in Methanol (*r*^2^)	Calibration Linearity in Steroid-Free Serum (*r*^2^)
STD	Tauroursodeoxycholic acid (TUDCA)	d4-GCA	498.28	2.58	80.00	60	0.998387	0.999016
STD	Taurocholic acid (TCA)	d4-TCA	514.28	2.7	80.00	64	0.999312	0.999820
STD	Glycoursodeoxycholic acid (GUDCA)	d4-GCA	448.28	2.92	74.00	35	0.997775	0.999103
STD	Glycocholic acid (GCA)	d4-GCA	464.28	3.06	74.00	34	0.998718	0.999051
STD	Taurochenodeoxycholic acid (TCDCA)	d4-GCA	498.28	3.3	80.00	60	0.997292	0.998966
STD	Taurodeoxycholic acid (TDCA)	d4-GCA	498.28	3.46	80.00	60	0.998165	0.998822
STD	Cholic acid (CA)	d4-CA	407.28	3.64	343.28	34	0.999410	0.999722
STD	Ursodeoxycholic acid (UDCA)	d4-UDCA	391.28	3.65	391.28	16	0.999290	0.999731
STD	Glycochenodeoxycholic acid (GCDCA)	d4-CA	448.28	3.67	74.00	35	0.998938	0.999806
STD	Hyodeoxycholic acid (HDCA)	d4-CA	391.28	3.77	391.28	16	0.999384	0.999745
STD	Glycodeoxycholic acid (GDCA)	d4-CA	448.28	3.87	74.00	35	0.998329	0.999870
STD	Taurolithocholic acid (TLCA)	d4-CDCA	482.28	4.02	80.00	60	0.997170	0.999418
STD	Chenodeoxycholic acid (CDCA)	d4-CDCA	391.28	4.41	391.28	16	0.999702	0.999748
STD	Glycolithocholic acid (GLCA)	d4-DCA	432.28	4.49	74.00	35	0.999886	0.998580
STD	Deoxycholic acid (DCA)	d4-DCA	391.28	4.51	391.28	16	0.999446	0.999799
IS	Taurocholic acid-d4 (d4-TCA)		518.28	2.7	80.00	64		
IS	Glycocholic acid-d4 (d4-GCA)		468.28	3.06	74.00	34		
IS	Cholic acid-d4 (d4-CA)		411.28	3.64	347.28	34		
IS	Ursodeoxycholic acid-d4 (d4-UDCA)		395.28	3.65	395.28	16		
IS	Chenodeoxycholic acid-d4 (d4-CDCA)		395.28	4.41	395.28	16		
IS	Deoxycholic acid-d4 (d4-DCA)		395.28	4.51	395.28	16		

**Table 2:** Serum concentrations of individual bile acids (BA) in healthy adult population.

BA Species	Mean (ng/mL)	2.5 Percentile (ng/mL)	97.5 Percentile (ng/mL)
Total BA	795.4	143.2	2152.4
TUDCA	<5	<5	5.2
GCDCA	203.5	24.8	706.5
HDCA	6.9	<5	14.1
GDCA	75.3	7.2	273.9
TLCA	<5	<5	<5
CDCA	97.4	5.1	521.3
GLCA	5.8	<5	21.7
DCA	161.4	9.6	542.8
GUDCA	40.1	3.9	145.8
TCA	11.3	<5	65.9
GCA	77.4	8.6	415.1
TCDCA	24.1	<5	92.8
TDCA	11.7	<5	45.1
UDCA	17.9	<5	77.7
CA	71.3	<5	495.0
